# Continuous Renal Replacement Therapy With Adsorbing Filter oXiris in Acute Kidney Injury With Septic Shock: A Retrospective Observational Study

**DOI:** 10.3389/fmed.2022.789623

**Published:** 2022-04-08

**Authors:** Mingjing Guan, Hao Wang, Xin Tang, Yuliang Zhao, Fang Wang, Ling Zhang, Ping Fu

**Affiliations:** ^1^Division of Nephrology, Kidney Research Laboratory, West China Hospital of Sichuan University, Chengdu, China; ^2^Division of Osteopathic, Department of Surgery Medicine, West China Hospital of Sichuan University, Chengdu, China

**Keywords:** sepsis/septic shock, continuous renal replacement therapy, acute kidney injury, oXiris, endotoxin

## Abstract

**Background and Objective:**

Sepsis/septic shock-associated acute kidney injury (S-AKI) is associated with prolonged kidney recovery and extremely high mortality. Extracorporeal blood purification therapy for the removal of endotoxin and cytokines might benefit patients with S-AKI. The purpose of this study was to compare the efficacy of adsorbing filter oXiris in the treatment of S-AKI.

**Design, Setting, Participants, and Measurements:**

This was a retrospective observational study conducted from September 2017 to June 2020 in ICU. All patients received CRRT for ≥24 h. The primary outcomes were mortality. The secondary outcomes included cardiovascular SOFA score and vasoactive-inotropic score (VIS), the SOFA, the reduction of inflammatory mediators.

**Results:**

A total of 136 septic shock patients with AKI were included. The interventional group (oXiris group; *n* = 70) received CRRT with endotoxic and cytokine adsorption function hemofilter (oXiris), while the control group (ST150 group; *n* = 66) was treated with the ST150 hemofilter. The early mortality in 7 and 14 days was significantly lower in oXiris group compared with ST150 group (7 days: 47.1 vs. 74.2%, *P* = 0.007; 14 days: 58.5 vs. 80.3%, *P* = 0.005), but the difference was not significant in 90-day mortality (71.4 vs. 81.8%, *P* = 0.160). Additionally, the reduction of the SOFA score in the oXiris group at 24, 48, and 72 h CRRT was significantly faster than that in the controlled group. Meanwhile, the reduction of VIS score in the oXiris group compared with the ST150 group at 24 and 48 h after the initiation of CRRT was statistically significant (*P* < 0.05). Furthermore, the decreases in procalcitonin were greater in the oXiris group than those in the ST150 group at 24, 48, and 72h after initiation of CRRT. Multivariate Cox regression model demonstrated that oXiris (vs. ST150) played a favorably important role in the prognosis of septic shock patients with a hazard ratio (HR) of 0.500 (95% CI: 0.280–0.892; *P* = 019).

**Conclusion:**

Although no difference was found in 90-day mortality, oXiris might reduce the short-term (<14-day) mortality compared with ST150 groups in septic shock with AKI. Further investigation in randomized controlled trials or high-quality prospective studies is warranted to validate the present findings.

## Introduction

Acute kidney injury (AKI), the most common complication of sepsis/septic shock in patients admitted to the intensive care unit (ICU) (1), is associated with high morbidity and mortality rates. The occurrence rate of sepsis/septic shock-associated AKI (S-AKI) ranges from 11 to 42% (2). However, this rate exceeds 50% in patients with severe AKI, resulting in higher mortality and prolonged kidney recovery (3). Although the mechanisms involved in S-AKI are not well established, they may involve the following aspects: immunological and autonomic dysregulation, macrovascular and microvascular dysfunction, and abnormal cellular response. Immunological and autonomic dysregulation comorbid with high levels of circulating endotoxin and cytokines accelerating the progression of S-AKI plays an important role (4). The development of S-AKI, caused by immunological dysregulation, is typically divided into two stages. In stage 1 (infection stage), pathogens are recognized by the immune system. The specific components on the surface of pathogens, such as endotoxin—a cell wall component of Gram-negative bacteria (GNB)—are termed pathogen-associated molecular patterns. These can be released into the blood circulation after bacterial lysis. During infection, pathogen-associated molecular patterns are first recognized by receptors expressed on the surface of immune cells (5). This signal activates leukocytes and induces the synthesis and release of pro-inflammatory and anti-inflammatory cytokines, including tumor necrosis factor-alpha (TNF-α), interleukin-1 (IL-1), IL-6, IL-8, and IL-10. The release of a large amount of cytokines in the blood circulation is termed “cytokine storm,” and is the primary cause of multiple organ dysfunction (5, 6). In stage 2, damaged host cells express damage-associated molecular patterns, such as high-mobility-group-box-1 (HMGB1), on their surfaces. Damage-associated molecular patterns are released into the blood circulation and recognized by pattern recognition receptors, which enhance leukocyte activation and cytokine synthesis, and aggravate the progression of uncontrolled immune inflammation (7). Immunization-induced paralysis occurs after the initial cytokine storm (8).

The treatment of S-AKI mainly involves appropriate antimicrobial therapy, initial fluid resuscitation, vasopressor support, and nutritional support. Extracorporeal blood purification therapy for the removal of endotoxin and cytokines is suggested as an adjuvant therapy for sepsis (9). However, there is a lack of positive multicenter randomized controlled trials (RCTs) confirming the currently available clinical evidences (10).

Presently, the hollow-fiber AN69 blood purification device (oXiris; Baxter, IL, United States) containing a higher amount of free positively charged amino groups polyethylenimine grafting has shown high adsorptive competence for endotoxin, cytokines, and other inflammatory mediators. Also, it has exhibited functioning antithrombogenic properties with membrane pregrafted 4,500 UI/m^2^ heparin and renal replacement therapy (RRT) (11). Currently, some case series have reported encouraging results (12, 13). However, many uncertainties remain around the clinical application of oXiris in patients with S-AKI due to the lack of large prospective studies in Asian patients (14). The purpose of this study was to compare the effectiveness of the oXiris hemofilter for the adsorption of endotoxin and cytokine and conventional hemofilter in patients with S-AKI.

## Materials and Methods

### Trial Design and Setting

This was a retrospective single-center, observational study conducted from September 2017 to June 2020 in West China Hospital of Sichuan University ICU (Chengdu, China). The study was approved by the Institutional Research Ethics Committees of West China Hospital of Sichuan University (Approval No. of the ethics committee: 2020-809), and inquirement for informed consent.

### Participants

From September 2017 to June 2020, patients with S-AKI due to Gram-negative bacteria or suspected GNB infection were involved. Continuous venovenous hemofiltration (CVVHDF) using oXiris haemofilter was performed. The patients’ cardiovascular SOFA, disease type and inclusion criteria matched those of a series of selected historical controls who had been treated with CVVHDF using ST150 hemofilter during the same period.

Patients who met the following criteria were eligible to participate: (I) CRRT ≥ 24 h; (II) septic shock; (III) AKI stage 2 or 3; (IV) cardiovascular SOFA score ≥ 3; (V) sepsis due to GNB infection (or suspected GNB infection). Patients with the following characteristics were excluded: (I) AKI associated with chronic kidney disease; (II) immunosuppressive treatment or steroids (prednisone > 0.5 mg/kg/day or equivalent); (III) autoimmune disorder; (IV) coexisting illness with a high probability of death (<6 months); (V) pregnancy; (VI) other modalities of blood purification were used; and (VII) inclusion in another ongoing study within the last 30 days.

Patients with S-AKI (age: 18–75 years; weight: >30 kg) were enrolled. The Third International Consensus Definitions for Sepsis and Septic Shock (Sepsis-3) criteria were used for diagnosis (15). The diagnosis and staging of AKI were determined using the 2012 Kidney Disease Improving Global Outcomes guidelines (16).

### Study Interventions

All patients were treated with continuous venovenous hemodiafiltration using a hemofilter, i.e., oXiris (Baxter) or ST150 (Baxter), through a Prismaflex CRRT set (Baxter). Blood flow rates were maintained between 150 and 200 mL/min. The therapeutic dosage was 30–35 mL/kg/h. Heparin and saline priming were applied in two cycles, respectively. Bicarbonate replacement solution (Chengdu Qingshan Likang Pharmaceutical Co., Ltd., Chengdu, China) was administered through a post-dilution method (replacement fluid/dialysate 1:1). Furthermore, the oXiris hemofilter was replaced every 12–24 h, typically after completing 72 h of treatment with oXiris, and then ST150 hemofilter was replaced when patients became stable. For anticoagulation, unfractionated or low molecular weight heparin or regional citrate anticoagulation were utilized. For vascular access, the femoral vein was selected, and a double-lumen 13 F hemodialysis catheter (Baxter) was inserted. Termination of CRRT was performed according to recent studies, that the urine output and serum creatine (SCr) were indicative of kidney recovery (17), vasopressor cessation, increased urine output ≥500 mL/24 h (without diuretics), correction of fluid overload, hemodynamic stability, and the possible need to shift to intermittent hemodialysis due to imminent discharge from the ICU (18).

### Data Collection

The follow-up period was 90 days. Clinical data were recorded and collected from the electronic medical records system at baseline, as well as at 24, 48, and 72 h after the initiation of CRRT. The following data were recorded: (1) demographic and clinical characteristics, including age, sex, sites of infection and pathogens, stage of AKI, SOFA score, cardiovascular SOFA score, vasoactive-inotropic score (VIS), filter sessions and duration of use, length of stay in the hospital and ICU, CRRT and hospital expenses, and recovery of kidney function and (2) mean arterial pressure (MAP), mechanical ventilation, some important laboratory measures, such as the hemoglobin, leukocytes (white blood cells), urea (blood urea nitrogen), SCr, C-reactive protein (CRP), IL6, procalcitonin (PCT), hydrogen ion concentration (pH), and lactate.

### Assessment Prognosis of Kidney Function

The prognosis of kidney function itemized as complete recovery, partial recovery, and dependence on dialysis. Complete recovery of kidney function was defined as a return to normal levels of SCr and a normal urine test after 90 days of follow-up. Partial recovery of kidney function was defined as no return to normal levels of SCr and/or persistent proteinuria and hematuria after 90 days of follow-up, without the need for hemodialysis. Dependence on dialysis was defined as the need for hemodialysis after 90 days of follow-up (19).

### Statistical Analysis

Normally distributed continuous variables were expressed as the mean ± standard deviation and non-normally distributed continuous variables as median [interquartile range (25th and 75th percentiles)]. The Wilcoxon test or *t*-test was used for comparisons between two groups, as appropriate. Categorical variables were expressed as frequency with proportions and were analyzed using the chi-squared test or Fisher’s exact test. Survival analysis was performed using the Kaplan–Meier curve and the rates were compared using the log-rank test. In addition, univariate and multivariate Cox regression analysis were performed to evaluate whether filter type is an important prognostic factor. All statistical analyses were conducted using the SPSS software for Windows version 26.0 (IBM Corporation, Armonk, NY, United States). *P*-values <0.05 denoted statistically significant differences.

## Results

### General Characteristics

#### Enrollment and Follow-Up

**FIGURE 1:**
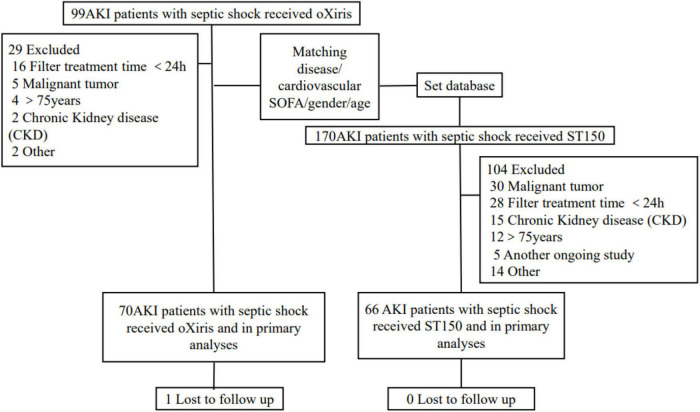
Patient recruitment, and flow of the study. AKI, acute kidney injure; SOFA, sequential organ failure assessment.

As shown in Figure 1, seventy patients with S-AKI who received CRRT with oXiris were included. The database was analyzed according to disease type, gender, age, and cardiovascular SOFA score matching to ST150 treatment of septic shock during the same period. In controlled group, 66 S-AKI patients receiving conventional CRRT (ST 150 hemofilter) fulfilled the eligibility matching criteria were included in the study finally. All included patients completed primary analysis except one patient from the oXiris group was lost to follow-up (90 days).

The general characteristics were as follows: the SOFA score was significantly higher in the oXiris group compared with the ST150 group (mean: 16.97 ± 3.56 vs. 14.98 ± 3.15, respectively; *P* = 0.001); the VIS score was significantly higher in the oXiris group compared with the ST150 group (median: 50.99[13.89–123.74] vs. 37.04[10.33–79.37], respectively; *P* = 0.038). Other general characteristics of these patients, including age, sex, body mass index, AKI stage, underlying diseases, comorbidity, peripheral arterial oxygen saturation to the inspired fraction of oxygen (SPO_2_/FiO_2_), mechanical ventilation, MAP, combined with ECMO were comparable between the two groups (all *P*-values > 0.05; as shown in Table 1).

**TABLE 1:** The general characteristics.

Variables	ST150 (*n* = 66)	oXiris (*n* = 70)	*P*-value
**Demographics**			
Age, year	54.42 ± 12.75	56.37 ± 15.06	0.420
BMI, kg/m^2^	24.49 ± 4.57	23.45 ± 4.17	0.170
Sex, (*n*, %)			0.350
Men	50(75.8)	48(68.6)	
Women	16(24.2)	22(31.4)	
**KDIGO AKI stage before CRRT initiation**			0.235
Stage 1	0(0.0)	0(0.0)	
Stage 2	11(16.7)	18(25.7)	
Stage 3	55(83.3)	52(74.3)	
**Underlying Diseases**			
Hypertension	10(15.2)	6(8.6)	0.234
Diabetes mellitus	14(21.2)	11(15.7)	0.408
Chronic obstructive pulmonary disease	3(4.5)	2(2.9)	0.601
Cardiovascular disease	1(1.5)	3(4.3)	0.339
Liver cirrhosis	2(3.0)	3(4.3)	0.697
**Comorbidity (n, %)**			
Cerebral edema	10(15.2)	3(4.3)	0.070
Respiratory failure	64(97.0)	64(91.4)	0.170
Heart failure	58(87.9)	59(84.3)	0.546
Acute liver injury	60(90.9)	61(87.1)	0.483
Abnormal coagulation function	59(89.4)	61(87.1)	0.684
Gastrointestinal bleeding	45(68.2)	38(54.3)	0.097
Thrombocytopenia	60(90.9)	59(84.3)	0.243
Anemia	63(95.5)	61(87.1)	0.088
Hypoproteinemia	64(97.0)	63(90.0)	0.102
Electrolyte disorder	64(97.0)	58(82.9)	0.060
**Respiratory and circulatory support**			
SPO_2_/FiO_2_	193(125–250)	198(153.85–250)	0.720
Mechanical ventilation, d	8(2.75–17.5)	8(4–15.25)	0.650
MAP, mmHg	86(77.75–94.17)	83(73.5–90.6)	0.140
Combined with ECMO (n, %)	6(9.0)	3(4.2)	0.260
VIS sore	37.04(10.33–79.37)	50.99(13.89–123.74)	0.038
**Total SOFA score on starting CRRT**			
Respiration	3.00(3.00–4.00)	3.00(3.00–4.00)	0.658
Coagulation	2.00(0.00–3.00)	2.50(1.00–3.00)	0.224
Liver	1.00(0.00–2.00)	2.00(0.00–2.00)	0.036
Cardiovascular	4.00(3.00–4.00)	4.00(4.00–4.00)	0.067
Renal	2.50(1.00–4.00)	4.00(2.00–4.00)	0.059
Central nervous system	4.00(3.00–4.00)	4.00(3.00–4.00)	0.996
Total SOFA	14.98 ± 3.15	16.97 ± 3.56	0.001

*BMI, body mass index; CRRT, continuous renal replacement therapy; MAP, mean arterial pressure; KDIGO, Kidney Disease: Improving Global Outcomes; AKI, acute kidney injury; SPO_2_/FiO_2_, arterial oxygen tension/inspired oxygen fraction; ECMO, extracorporeal membrane oxygenation; VIS, vasoactive-inotropic score=dopamine dose (μg/kg/min) + dobutamine dose (μg/kg/min) + 100 × adrenaline dose (μg/kg/min) + 100 × noradrenaline dose (μg/kg/min) + 10 × milrinone dose (μg/kg/min) + 10.000 × vasopressin dose (U/kg/min); SOFA, sequential organ failure assessment.*

#### Baseline Laboratory Data

Laboratory data suggested that the respective baseline levels coagulation function index, the value of activated partial thromboplastin time, was worse in the oXiris group versus the ST150 group (median: 51.10[37.35–71.80] vs. 37.10[32.70–55.60] s, respectively, *P* = 0.017). Meanwhile, the baseline levels of IL-6 and PCT in the oXiris group were higher than those recorded in the ST150 group (median: 515.15[103.00–5000.00] vs. 179.55[43.65–1158.50] pg/mL, respectively, *P* = 0.048) and(median: 31.40 [5.63–79.40] vs. 5.46[2.00–28.86] ng/mL, respectively, *P* < 0.001) (Table 2).

**TABLE 2:** The laboratory data.

Variables	ST150 (*n* = 66)	oXiris (*n* = 70)	*P*-value
Hemoglobin (g/L)	97.00(80.00–115.00)	85.00(77.00–105.00)	0.078
Hematocrit (g/g)	0.30(0.24–0.36)	0.27(0.24–0.31)	0.091
Leukocyte (10^9^ /L)	12.81(8.05–16.55)	10.88(6.04–19.38)	0.864
Granulocyte (%)	84.17 ± 11.85	85.19 ± 11.58	0.614
Platelet (10^9^/L)	75.00(36.00–139.00)	52.00(25.00–106.00)	0.094
Prothrombin time (s)	16.50(14.10–21.70)	17.35(15.00–22.10)	0.255
Actived partial thrombolastin time (s)	37.10(32.70–55.60)	51.10(37.35–71.80)	0.017
international normalized ratio	1.49(1.24–2.00)	1.60(1.36–2.03)	0.309
Fibrinogen (g/L)	3.07(1.58–4.35)	2.37(1.54–4.01)	0.468
D-dimer	9.26(4.70–17.85)	9.97(3.59–17.09)	0.577
Total bilirubin (umol/L)	27.30(17.60–65.50)	40.10(20.40–75.70)	0.164
Alanine aminotransferase (IU/L)	36.00(19.00–179.00)	54.00(19.00–174.00)	0.602
Total protein (g/L)	52.65(46.40–59.40)	49.30(44.60–56.90)	0.065
Albumin (g/L)	29.65(26.40–33.50)	30.10(26.10–33.70)	0.695
Aspartate aminotransferase (IU/L)	77.00(40.00–261.00)	102.00(35.00–359.00)	0.876
TnT (ng/mL)	88.05(40.40–253.50)	198.80(37.50–568.20)	0.220
Blood pH	7.31(7.25–7.37)	7.33(7.26–7.39)	0.393
lactate mmol/L	3.15(2.00–6.10)	4.20(2.20–7.70)	0.181
**Inflammatory index**			
C-reactive protein mg/L	120.00(73.45–194.00)	162.00(91.10–258.00)	0.181
Interleukin-6 pg/mL	179.55(43.65–1158.50)	515.15(103.00–5,000.00)	0.048
Procalcitonin ng/mL	5.46(2.00–28.86)	31.40(5.63–79.40)	<0.001

*TnT, troponin T; pH, potential of hydrogen.*

#### Conditions of the Infection

The majority of patients suffered from pulmonary sepsis, followed by intra-abdominal sepsis. Positive blood culture was significantly different between the oXiris group and ST150 group (32 [45.7%] vs. 10 [15.2%], respectively, *P* < 0.001). The presence of more than three infection sites was significantly more common in the oXiris group vs. the ST150 group (21 [30.0%] vs. 9 [13.6%], respectively, *P* = 0.021). Pathogenic bacteria were mainly GNB, with *Acinetobacter baumannii* and *Klebsiella* being the major bacterial species (Table 3).

**TABLE 3:** The conditions of the infection.

Variables	ST150 (*n* = 66)	oXiris (*n* = 70)	*P*-value
**Site of infection**			
Pulmonary	57(86.4)	58(82.9)	0.572
Intra-abdomen	30(45.5)	26(37.1)	0.325
Skin or tissue	7(10.6)	9(12.9)	0.684
Urinary	11(16.7)	8(11.4)	0.379
Blood	10(15.2)	32(45.7)	< 0.001
Cardiac valves myocardium	0(0.0)	4(5.7)	0.049,
Unknown	5(7.6)	3(4.3)	0.415
One site or less	23(34.8)	22(31.4)	0.672
Two sites	34(51.5)	27(38.6)	0.129
Three sites or more	9(13.6)	21(30.0)	0.021
**Culture-proven infection**			
Gram positive	46(69.7)	54(77.1)	0.325
Gram negative	12(18.2)	12(17.1)	0.874
Gram positive and negative	8(12.1)	9(12.9)	0.897
Fungal infection	19(28.8)	17(24.3)	0.552
Virus infection	4(6.1)	1(1.4)	0.151
**Pathogen**			
*Staphylococcus aureus*	6(9.1)	3(4.3)	0.460
*Pseudomonas aeruginosa*	11(16.7)	8(11.4)	0.592
*Acinetobacter baumannii*	22(33.3)	23(32.9)	0.310
*Escherichia coli*	12(18.2)	4(5.7)	0.106
*Klebsiella*	22(33.3)	11(15.7)	0.098
*Candida Albicans*	8(12.1)	11(15.7)	0.560

### Primary Endpoint

**FIGURE 2:**
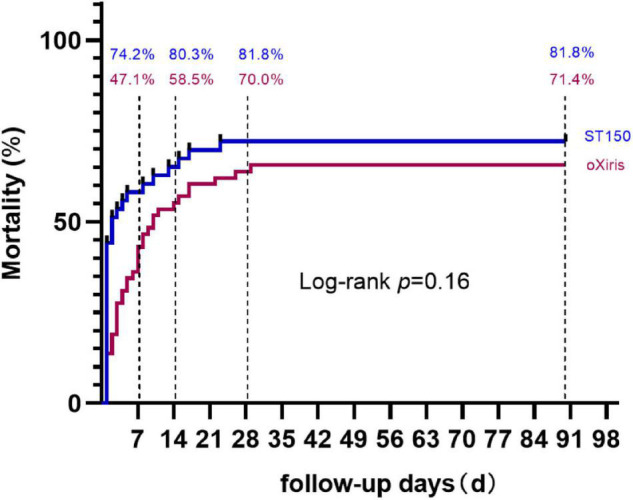
The Kaplan–Meier estimates of mortality at 90 days. In the modified intention-to-treat analysis, death at 90 days occurred in 50 patients (71.4%) in the group that received oXiris and in 54 (81.8%) in the group that received ST150. *P* = 0.16 for the between-group difference in the Kaplan–Meier time-to-event analysis.

As shown in Figure 2, the mortality rate was lower in the oXiris group than that in the ST150 group; nevertheless, the difference was insignificant between the two groups. There were 50 patients (71.4%) and 44 patients (81.8%) dead at day 90, respectively in the oXiris and ST150 groups (*P* = 0.160) (Figure 2).

On the other hand, significant differences were observed in mortality at day 7 and day 14 between the oXiris and ST150 group (day 7: 47.1 vs. 74.2%, respectively, *P* = 0.007; day 14: 58.5 vs. 80.3%, respectively, *P* = 0.005). However, there was no significant difference in mortality at day 28 (Figure 2).

### Secondary Endpoints

**FIGURE 3:**
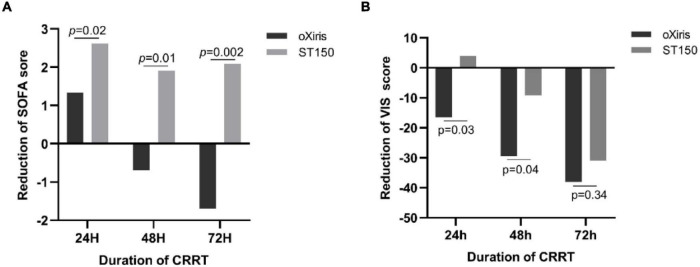
The Reduction of SOFA and VIS score in treatment period (0–72 h) in the oXiris filter groups and ST150 groups. Panel **(A)** shows the median at Reduction of SOFA each time point and Panel **(B)** shows the median change reduction of VIS.

After 24, 48, and 72 h of CRRT, the SOFA score of patients from the oXiris group decreased faster compared with that of patients from the ST150 group. There were group differences for mean change from baseline on SOFA score (at 24 h: 1.3 ± 3.34 vs. 2.6 ± 2.73, *P* = 0.020; 48 h: −0.7 ± 5.89 vs. 1.9 ± 3.74, *P* = 0.010; and 72 h: −1.7 ± 5.99 vs. 2.1 ± 3.58, *P* = 0.002, respectively). Furthermore, the difference in the reduction of the VIS score between two groups was also statistically significant (at 24 h: −16.44 [−81.54–5.42] vs. 3.96 [−35.06–25.17], *P* = 0.030; and 48 h: −29.41 [−61.95—3.39] vs. −9.26 [−49.38–22.22], *P* = 0.044, respectively) (Figure 3). There was no significant difference cardiovascular SOFA (*P* > 0.05) between the two groups at 24, 48, and 72 h after CRRT.

**FIGURE 4:**
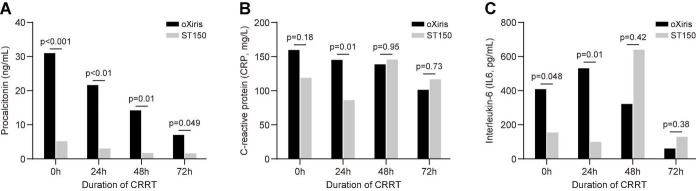
Blood inflammatory mediators during filter treatment period (0–72 h) in the oXiris filter and ST15 groups. **(A)** Procalcitonin, **(B)** C-reaction protein (CRP), **(C)** interleukin (IL)-6.

As shown in Figure 4, in terms of the levels of inflammatory mediators, the decrease in PCT was higher in the oXiris group than that in the ST150 group at 24, 48, and 72 h after treatment (21.93 [4.43–48.33] vs. 3.3 [1.18–18.05] ng/mL, P < 0.010; 14.5 [3.0–31.7] vs. 2.0 [1.4–4.1] ng/mL, *P* < 0.01; and 7.3 [2.7–7.6] vs. 1.9 [1.2–7.2] ng/mL, *P* < 0.05, respectively). At 24 h following CRRT, the decrease in the levels of CRP and IL-6 in the oXiris group was higher than that noted in the ST150 group (CRP: 146.50 [110.25–259.00) vs. 87.50 [62.65–159.25] mg/L, *P* = 0.010; and IL-6: 537.00 [93.21–3851.00] vs. 105.00 [29.20–988.13] pg/mL, *P* = 0.010, respectively). However, there was no statistically significant difference at 48 and 72 h after CRRT.

**FIGURE 5:**
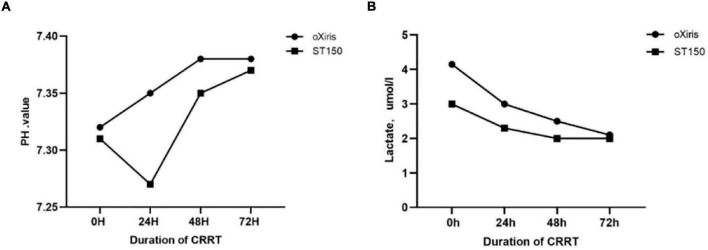
The homeostasis indicators during filter treatment period (0–72 h) in the oXiris filter and ST15 groups. **(A)** Hydrogen ion concentration (pH), **(B)** Lactate(Lac).

In addition, there was no significant difference in pH, and lactate (*P* > 0.05) between the two groups at 24, 48, and 72 h after CRRT (Figure 5).

A shorter required CRRT time was reported in the oXiris group vs. the ST150 group (48.00 [32.50–72.00] vs. 115.0 [52–240.0] h, *P* < 0.001, respectively). However, the number of hemofilter sessions was not significantly different between the two groups (3.50 [2.00–5.00] sets vs. 3.00 [1.00–6.00] sets, respectively, *P* = 0.474). Both the duration of hospital stay and ICU were not significantly different between the groups (duration of hospital stay: 22.00 [10.00–39.00] vs.19.50 [9.00–35.00] days, respectively, *P* = 0.589; duration of ICU stay: 14.00 [7.00–28.00] vs. 13.50 [7.00–26.00] days, respectively, *P* = 0.927). The cost of CRRT was higher in the oXiris group than that in the ST150 group (47,277.54 [28,690.08–774,444.00] vs. 23,419.60 [11,730.00–44105.76] CNY, respectively, *P* < 0.001). Nonetheless, there was no significant difference in hospital expenses between two groups (270,659.15 [137,109.40–431,402.01] vs. 200,190.80 [118,454.84–319,262.61] CNY, respectively, *P* = 0.065) (Table 4).

**TABLE 4:** The secondary outcomes.

Variables	ST150 (*n* = 66)	oXiris (*n* = 70)	*P*-value
Filter duration, h	115.00(52.00–240.00)	48.00(32.50–72.00)	<0.001
Filter sessions	3.00(1.00–6.00)	3.50(2.00–5.00)	0.474
Length of stay, mean, day			
Hospital	19.50(9.00–35.00)	22.00(10.00–39.00)	0.589
ICU	13.50(7.00–26.00)	14.00(7.00–28.00)	0.927
Expense of stay, median, CNY			
Expense of CRRT	23,419.61(11,770.00–44,105.76)	47277.54(28,690.08–74,444.00)	<0.001
Expense of hospital	200,190.80(118,454.84–319,262.61)	270,659.15(137,109.40–431,402.01)	0.065

*ICU, intensive care unit; CRRT, continuous renal replacement therapy.*

### Prognosis of Kidney Function

We assessed the prognosis of kidney kidney function in the two groups over a 90-day follow-up period. Patients who were lost to follow-up were considered non-chronic kidney disease patients. As shown in Table 5, 32 patients survived in the two groups (20 and 12 patients in the oXiris and ST150 groups, respectively). In the oXiris group, the kidney function of 19 patients (95.0%) returned to normal, whereas the creatinine levels of one patient (5.0%) did not return to normal without hemodialysis. In the ST150 group, the kidney function of 10 patients (83.4%) returned to normal, whereas that of two patients (16.6%) did not; moreover, one patient (8.3%) developed chronic kidney disease that required intermittent hemodialysis. After treatment for 90 days, the number of patients with recovery of kidney function was not significantly different between the two groups (*P* = 0.540) (Table 5).

**TABLE 5:** The prognosis of kidney function.

Kidney function recovery	oXiris group (*n* = 70)	ST150 group (*n* = 66)	*P*-value
Complete recovery of kidney function	19(95.0)	10(83.4)	0.540
Partial recovery of kidney function	1(5.0)	1(8.3)	
Dialysis-dependent	0(0.0)	1(8.3)	

To determine whether the filter type affected the prognosis of patients, we performed the univariate and multivariate COX analysis. The univariate COX analysis result showed that filter type oXiris (vs. ST150) was significantly correlated with 90-day outcomes of patients, with HR = 0.640 (95%CI: 0.434–0.943; *P* = 0.024). After adjusting the general characteristics (pre-treatment VIS score, liver SOFA and total SOFA score) the filter type was also the factor affecting the outcome (*P* = 0.022). Similarly, we further adjusted the remaining variables based on Model 2 (plus Laboratory data and conditions of the infection) and the results also showed that filter type was significantly corrected with the prognosis of septic shock with AKI. Compared with group ST150, patients in group oXiris had a lower risk of death than those in group ST150, HR = 0.500 (95% CI: 0.280–0.892) (Table 6).

**TABLE 6:** Cox regression analysis of variables associated with 90-day outcomes of patients.

Model	HR (95%CI)	*P*-value
**Model 1**		
oXiris (vs. ST150)	0.640(0.434–0.943)	0.024
**Model 2**		
oXiris (vs. ST150)	0.620(0.412–0.933)	0.022
VIS sore	1.003(1.000–1.006)	0.085
Liver SOFA	1.100(0.890–1.360)	0.376
Total SOFA	1.005(0.929–1.088)	0.892
**Model 3**		
oXiris (vs. ST150)	0.500(0.280–0.892)	0.019
VIS sore	1.004(1.001–1.008)	0.024
Liver SOFA	1.315(0.992–1.744)	0.057
Total SOFA	1.000(0.901–1.109)	0.995
Actived partial thrombolastin time (s)	1.005(0.998–1.012)	0.168
Interleukin-6	1.000(1.000–1.000)	0.164
Procalcitonin	0.993(0.983–1.003)	0.181
Blood	1.098(0.534–2.260)	0.799
Three sites or more	0.880(0.419–1.846)	0.735

*Adjust variables in Model 2 are the general characteristics, in Model 3 are the general characteristics, laboratory data and conditions of the infection.*

## Discussion

In the present study, a tendency toward lower mortality in the oXiris group compared with the ST150 group was observed after 90 days of follow-up among S-AKI patients requiring CRRT; however, the difference was not statistically significant. Statistically significant differences were recorded for 7 and 14 days mortality. The results suggested that oXiris can improve the early survival rate, endotoxin is an important factor in triggering inflammatory cascade reaction, and the increase of endotoxin level in patients with sepsis will lead to an increase in mortality. oXiris can eliminate endotoxin in sepsis patients in the early stage, so as to improve the hemodynamics and early prognosis of sepsis patients. We also found that the VIS score improved faster in the oXiris group versus the ST150 group during the duration of CRRT. Moreover, oXiris offered a potential advantage in decreasing the levels of inflammatory mediators compared with ST150.

Treatment of sepsis with extracorporeal blood purification technology has long been a field of research interest. At present, several extracorporeal blood purification devices aiming at the removal of endotoxin and cytokines in kidney support and immunomodulation therapy are available (18). The polymyxin B-immobilized fiber column (Toraymyxin^®^; Toray, Tokyo, Japan) is the most widely used specific endotoxin removal therapy in Japan for patients with GNB infection. However, this method lacks the ability to capture inflammatory mediators and cytokines. Thus far, numerous clinical research studies failed to demonstrate any improvement on mortality. Nonetheless, recent RCT results suggest that patients with severe sepsis and circulating endotoxin levels between 0.6 and 0.9 EU/mL may benefit from this approach (20). The Alteco^®^ LPS adsorber (Alteco Medical AB, Lund, Sweden) utilized a synthetic peptide to cover the surface of a porous polyethylene matrix for the adsorption of endotoxin. However, a multicenter RCT [ASSET (abdominal septic shock-endotoxin adsorption treatment)] evaluating this method was terminated ahead of time due to patient recruitment problems (21). The CytoSorb^®^ device (CytoSorbents Corporation, Monmouth Junction, NJ, United States) with porous adsorbent polymer beads could adsorb excessive inflammatory mediators and cytokines, but not endotoxin. A previous *in vitro* experiment showed a cytokine removal rate exceeding 90–95% (22, 23). Nevertheless, clinical studies remain scarce and only some case series have demonstrated an amelioration of hemodynamic parameters and lactate levels (24, 25).

The hollow-fiber AN69 blood purification device oXiris containing a higher amount of free positively charged amino groups polyethylenimine grafting achieves highly adsorptive competence for endotoxin, cytokines, and other inflammatory mediators simultaneously. In addition, it exhibits functioning antithrombogenic properties with membrane pregrafted 4,500 UI/m^2^ heparin, which can reduce the occurrence of thrombosis, prolong the life of the hemofilter, and improve the efficiency of CRRT. *In vitro* and clinical studies confirmed that oXiris can significantly reduce the levels of PCT, endotoxin, inflammatory mediators, and cytokines (22, 26, 27). The decrease in endotoxin, inflammatory mediators, and cytokines was associated with the reduction in the dosage of vasopressor and improvement of the SOFA score (28, 29). This study demonstrated that there was no significant difference between the two groups in MAP during treatment. However, evaluation of the use of vasopressors in the two groups of patients showed that a reduction in the use of vasopressor drugs was helpful in maintaining hemodynamic stability. The cardiovascular SOFA score was assessed based on the dosage of norepinephrine, epinephrine, dopamine. Due to the presence of numerous critical illnesses, the mortality rate was extremely high, and the final cardiovascular SOFA score was not significantly different between the two groups. However, there was a significant reduction in the SOFA score in the oXiris group. We hypothesized that the specific adsorption of endotoxins, inflammatory media, and cytokines may improve other SOFA scores, such as the coagulation and hepatic SOFA scores. oXiris showed a specific capacity for adsorbing cytokines. Although the change was not significantly different, the amplitude of PCT reduction in the oXiris group had a significant advantage compared with that recorded in the ST150 group.

Some clinical studies also found that oXiris could improve hemodynamic parameters, reduce the demand for norepinephrine, increase the lactate clearance rate, and decrease the requirement for resuscitation fluid (30, 31). This study also found that the condition was severer in the oXiris group during CRRT. Moreover, the SOFA score, VIS score, improved in the oXiris group during treatment, exceeding those recorded in the ST150 group. After 72 h of treatment, the metabolic state of patients had significantly improved.

Previous studies have shown that the incidence of AKI in patients with septic shock was approximately 50% and related to a high mortality rate (3). In this study, the mortality among severe patients with septic shock and AKI was markedly high; this may be explained by the fact that CRRT was used as remedial treatment in most cases. Patients often have multiple sites and multiple complicated bacterial infections, especially multi-resistant bacterial infections, leading to high SOFA scores and high mortality. There was no significant difference in 90-day mortality between the two groups; however, there was a significant difference in mortality at day 7 and 14. These findings suggested that oXiris may improve early clinical outcomes. In this study, sepsis was mainly caused by Gram-negative pathogens; nevertheless, we were unable to compare the mortality rates of patients by type of pathogens due to the limited case number and existence of multiple bacterial infections.

Currently, there is no reference consensus regarding the application of oXiris. Recently, a multi-country RCT showed better kidney function at the beginning of treatment with oXiris among critically ill patients with AKI (stages 2–3). Early initiation of treatment can shorten the duration of CRRT and length of ICU stay (32). Based on the available evidence, it is suggested that early initiation of CRRT may be helpful in the recovery of kidney function in patients with S-AKI. In the oXiris group of our study, the median number of filters used was 3.5, and the actual treatment time was <72 h. Considering that the number of filters used may affect the treatment effect, oXiris may be saturated after 24 h of treatment; therefore, it is reasonable to replace these filters every 24–48 h (18). There was no significant difference in the number of filters used between the two groups. Future research should focus on the timing of initiation, optimal duration of use and indication of filter exchange when applying oXiris.

Antibiotics are of vital importance in patients with septic shock. It is established that, apart from the adsorption of endotoxin and inflammatory mediators, hemofilter membranes are also able to capture antibiotics. Currently, there are no relevant guidelines for the adjustment of antibiotic dosage during CRRT, and the effective concentration of antibiotics has not been determined. Notably, use of the hemofilter such as oXiris may further lower the effective concentration of the antibiotic, potentially accelerating the deterioration of patient’s condition, which warrants clinical observation in the future

This study had several limitations. Firstly, this was a single-center observational study, and the sample size was small. Secondly, the baseline levels were uneven, the disease degree of the two groups of patients did not match completely, and the status of illness was severer in the oXiris group. Ultimately, the central nervous system SOFA may have been overestimated due to the invasive ventilatory support therapy administered to patients who were mostly in a state of sedation and analgesia.

## Conclusion

Although no difference was found in 90-day mortality, oXiris might reduce the short-term (<14-day) mortality compared with ST150 groups in septic shock with AKI. Randomized controlled trials or high-quality prospective studies are warranted to validate the present findings.

## Data Availability Statement

The original contributions presented in the study are included in the article/supplementary material, further inquiries can be directed to the corresponding author.

## Ethics Statement

The studies involving human participants were reviewed and approved by the Research Ethics Committee of the West China Hospital of Sichuan University. Since this article is a retrospective article, there was no need to sign an informed consent.

## Author Contributions

LZ and PF were mainly responsible for program design and modification. MG, HW, XT, YZ, and FW were involved in this clinical trial and vouch for the adherence of the trial to the protocol, for the accuracy of the data. MG and HW conducted the statistical analysis and wrote the first draft. All authors reviewed, revised, and approved the final version of the manuscript and agreed to the submission of this manuscript.

## Conflict of Interest

The authors declare that the research was conducted in the absence of any commercial or financial relationships that could be construed as a potential conflict of interest.

## Publisher’s Note

All claims expressed in this article are solely those of the authors and do not necessarily represent those of their affiliated organizations, or those of the publisher, the editors and the reviewers. Any product that may be evaluated in this article, or claim that may be made by its manufacturer, is not guaranteed or endorsed by the publisher.

## References

[1] Hoste EA Bagshaw SM Bellomo R Cely CM Colman R Cruz DN Epidemiology of acute kidney injury in critically Ill patients: the multinational AKI-EPI study. *Intensive Care Med.* (2015) 41:1411–23. 10.1007/s00134-015-3934-7 26162677

[2] Bagshaw SM George C Bellomo R Committee ADM. Early acute kidney injury and sepsis: a multicentre evaluation. *Crit Care.* (2008) 12:R47. 10.1186/cc6863 18402655 PMC2447598

[3] Peters E Antonelli M Wittebole X Nanchal R Francois B Sakr Y A worldwide multicentre evaluation of the influence of deterioration or improvement of acute kidney injury on clinical outcome in critically ill patients with and without sepsis at ICU admission: results from The Intensive Care Over Nations audit. *Crit Care.* (2018) 22:188. 10.1186/s13054-018-2112-z 30075798 PMC6091052

[4] Ronco C Bellomo R Kellum JA. Acute kidney injury. *Lancet.* (2019) 394:1949–64. 10.1016/S0140-6736(19)32563-2 31777389

[5] Angus DC van der Poll T. Severe sepsis and septic shock. *N Engl J Med.* (2013) 369:840–51. 10.1056/NEJMra1208623 23984731

[6] Ronco C Tetta C Mariano F Wratten ML Bonello M Bordoni V Interpreting the mechanisms of continuous renal replacement therapy in sepsis: the peak concentration hypothesis. *Artif Organs.* (2003) 27:792–801. 10.1046/j.1525-1594.2003.07289.x 12940901

[7] Zarbock A Gomez H Kellum JA. Sepsis-induced acute kidney injury revisited: pathophysiology, prevention and future therapies. *Curr Opin Crit Care.* (2014) 20:588–95. 10.1097/MCC.0000000000000153 25320909 PMC4495653

[8] Hotchkiss RS Monneret G Payen D. Immunosuppression in sepsis: a novel understanding of the disorder and a new therapeutic approach. *Lancet Infect Dis.* (2013) 13:260–8. 10.1016/S1473-3099(13)70001-X 23427891 PMC3798159

[9] Zhang L Feng Y Fu P. Blood purification for sepsis: an overview. *Precis Clin Med.* (2021) 4:45–55. 10.1093/pcmedi/pbab005 PMC8982546 35693122

[10] Cecconi M Evans L Levy M Rhodes A. Sepsis and septic shock. *Lancet.* (2018) 392:75–87. 10.1016/S0140-6736(18)30696-2 29937192

[11] Baxter. Data on file. oXiris. Instructions for use. (2017).

[12] Zhang L Yan Tang GK Liu S Cai J Chan WM Yang Y Hemofilter with adsorptive capacities: case report series. *Blood Purif.* (2019) 47:1–6. 10.1159/000499357 30982026

[13] Wei T Chen Z Li P Tang X Marshall MR Zhang L Early use of endotoxin absorption by oXiris in abdominal septic shock: a case report. *Medicine (Baltimore).* (2020) 99:e19632. 10.1097/MD.0000000000019632 32664051 PMC7360291

[14] Zhang L Cove M Nguyen BG Lumlertgul N Ganesh K Chan A. Adsorptive hemofiltration for sepsis management: expert recommendations based on the Asia Pacific experience. *Chin Med J (Engl).* (2021) 134:2258–60. 10.1097/CM9.0000000000001671 34402478 PMC8478384

[15] Singer M Deutschman CS Seymour CW Shankar-Hari M Annane D Bauer M The third international consensus definitions for sepsis and septic shock (Sepsis-3). *JAMA.* (2016) 315:801–10. 10.1001/jama.2016.0287 26903338 PMC4968574

[16] Khwaja A. KDIGO clinical practice guidelines for acute kidney injury. *Nephron Clin Pract.* (2012) 120:c179–84. 10.1159/000339789 22890468

[17] Viallet N Brunot V Kuster N Daubin D Besnard N Platon L Daily urinary creatinine predicts the weaning of renal replacement therapy in ICU acute kidney injury patients. *Ann Intensive Care.* (2016) 6:71. 10.1186/s13613-016-0176-y 27443673 PMC4956634

[18] Monard C Rimmelé T Ronco C. Extracorporeal blood purification therapies for sepsis. *Blood Purif.* (2019) 47:2–15. 10.1159/000499520 30974444

[19] Kidney Disease: Improving Global Outcomes (Kdigo) Ckd Work Group. KDIGO 2012 clinical practice guideline for the evaluation and management of chronic kidney disease. *Kidney Int Suppl.* (2013) 3:1–150. 10.1038/kisup.2012.73

[20] Dellinger RP Bagshaw SM Antonelli M Foster DM Klein DJ Marshall JC Effect of targeted polymyxin B hemoperfusion on 28-day mortality in patients with septic shock and elevated endotoxin level: the EUPHRATES randomized clinical trial. *JAMA.* (2018) 320:1455–63. 10.1001/jama.2018.14618 30304428 PMC6233793

[21] Lipcsey M Tenhunen J Sjolin J Frithiof R Bendel S Flaatten H Abdominal septic shock – endotoxin adsorption treatment (ASSET) – endotoxin removal in abdominal and urogenital septic shock with the Alteco^®^ LPS Adsorber: study protocol for a double-blinded, randomized placebo-controlled trial. *Trials.* (2016) 17:587. 10.1186/s13063-016-1723-4 27931259 PMC5146888

[22] Malard B Lambert C Kellum JA. In vitro comparison of the adsorption of inflammatory mediators by blood purification devices. *Intensive Care Med Exp.* (2018) 6:12. 10.1186/s40635-018-0177-2 29728790 PMC5935601

[23] Gruda MC Ruggeberg KG O’Sullivan P Guliashvili T Scheirer AR Golobish TD Broad adsorption of sepsis-related PAMP and DAMP molecules, mycotoxins, and cytokines from whole blood using CytoSorb^®^ sorbent porous polymer beads. *PLoS One.* (2018) 13:e0191676. 10.1371/journal.pone.0191676 29370247 PMC5784931

[24] Houschyar KS Pyles MN Rein S Nietzschmann I Duscher D Maan ZN Continuous hemoadsorption with a cytokine adsorber during sepsis – a review of the literature. *Int J Artif Organs.* (2017) 40:205–11. 10.5301/ijao.5000591 28525674

[25] Kogelmann K Jarczak D Scheller M Druner M. Hemoadsorption by CytoSorb in septic patients: a case series. *Crit Care.* (2017) 21:74. 10.1186/s13054-017-1662-9 28343448 PMC5366999

[26] Turani F Barchetta R Falco M Busatti S Weltert L. Continuous renal replacement therapy with the adsorbing filter oXiris in septic patients: a case series. *Blood Purif.* (2019) 47:54–8. 10.1159/000499589 30982024

[27] Broman ME Hansson F Vincent JL Bodelsson M. Endotoxin and cytokine reducing properties of the oXiris membrane in patients with septic shock: a randomized crossover double-blind study. *PLoS One.* (2019) 14:e0220444. 10.1371/journal.pone.0220444 31369593 PMC6675097

[28] Shum HP Chan KC Kwan MC Yan WW. Application of endotoxin and cytokine adsorption haemofilter in septic acute kidney injury due to Gram-negative bacterial infection. *Hong Kong Med J.* (2013) 19:491–7. 10.12809/hkmj133910 23650198

[29] Turani F Candidi F Barchetta R Grilli E Belli A Papi E Continuous renal replacement therapy with the adsorbent membrane oXiris in septic patients: a clinical experience. *Crit Care.* (2013) 17:63. 10.1186/cc12001

[30] Govil D Gupta S Srinivasan S Patel S Jagadeesh K Shafi M 055 cytokine removal in sepsis: does their levels co-relate with outcome. *Kidney Int Rep.* (2017) 2:S26. 10.1016/J.EKIR.2017.06.087

[31] Schwindenhammer V Girardot T Chaulier K Grégoire A Monard C Huriaux L oXiris^®^ use in septic shock: experience of two French centres. *Blood Purif.* (2019) 47:29–35. 10.1159/000499510 30982028

[32] Bagshaw SM Wald R Adhikari NKJ Bellomo R da Costa BR Dreyfuss D Timing of initiation of renal-replacement therapy in acute kidney injury. *N Engl J Med.* (2020) 383:240–51. 10.1056/NEJMoa2000741 32668114

